# Correction to: CircPSMC3 suppresses the proliferation and metastasis of gastric cancer by acting as a competitive endogenous RNA through sponging miR-296-5p

**DOI:** 10.1186/s12943-020-01252-z

**Published:** 2020-09-09

**Authors:** Dawei Rong, Chen Lu, Betty Zhang, Kai Fu, Shuli Zhao, Weiwei Tang, Hongyong Cao

**Affiliations:** 1 Department of General Surgery, Nanjing First Hospital, Nanjing Medical University, Nanjing, Jiangsu China; 2 Michael G. DeGroote School of Medicine, McMaster University, Hamilton, Ontario Canada; 3 Department of General Clinical Research Center, Nanjing First Hospital, Nanjing Medical University, Nanjing, Jiangsu China

**Correction to: Mol Cancer 18, 25 (2019)**

**https://doi.org/10.1186/s12943-019-0958-6**

After publication of the article [1], the authors reported errors of inter-duplication in Fig. 4f , Fig. 5g and Fig. 5h. The authors have confirmed that the “Transwell assay” images of “Mock+ miR-NC in AGS cells, circ-PSMC3+miR-296-5p in MGC803 cells, circ-PSMC3+miR-296-5p in AGS cells” group were mistakenly presented in the original Fig. 5h. This mistake was caused by that the folder was poorly managed and the same pictures are used unintentionally. The “Migration assay” image of “miR-NC of 24h in AGS cells” in Fig. 4f contained an inter-duplication in error with the image of “Mock+ miR-NC of 24h in MGC803 cells” in Fig. 5g, which was caused by the misplacement of the list of the “Migration assay” images in Fig. 5g.The histogram does not need to be changed, because we did not use the wrong picture when analyzing the original data, and the conclusion of this part is a negative result, which does not affect the overall conclusion of the article. The authors apologize for any inconvenience caused by unintentional misplace.

**Fig. 4:**
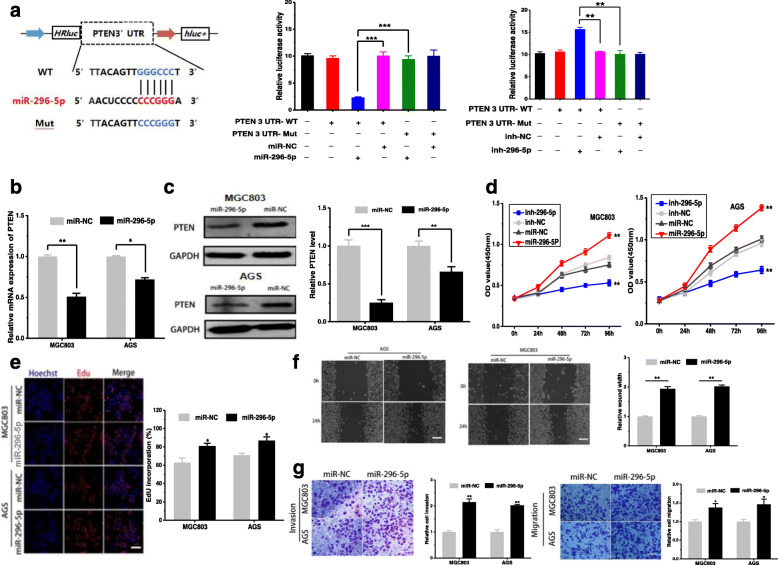
.

**Fig. 5:**
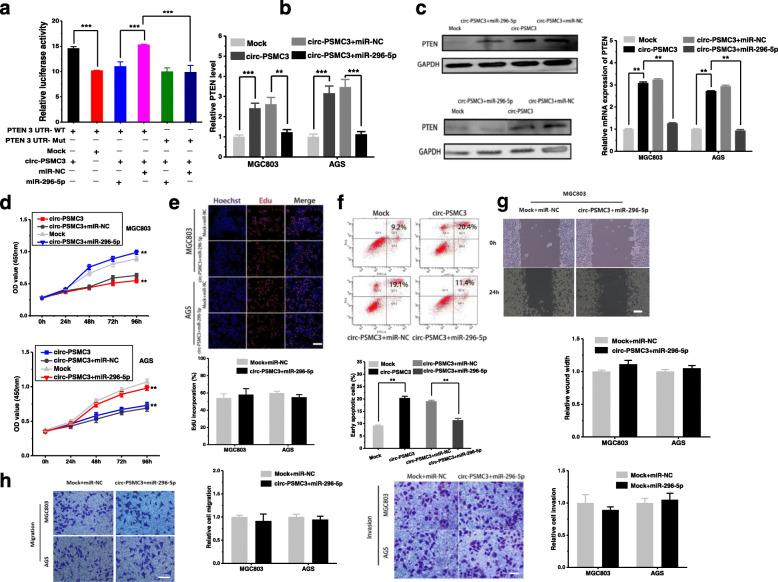
.

The correct figures are updated below.
